# Phosphaturic mesenchymal tumor of the popliteal fossa: a case report and literature review

**DOI:** 10.3389/fonc.2024.1501499

**Published:** 2024-12-19

**Authors:** Yingjie Wang, Shiwei Liu, Caixia Li, Wenjing Song, Yimin Zhang, Jun Wang

**Affiliations:** ^1^ Joint Surgery Department, Weifang People’s Hospital, Shandong Second Medical University, Weifang, Shandong, China; ^2^ Department of Oral Medicine, Weifang People’s Hospital, Shandong Second Medical University, Weifang, Shandong, China; ^3^ Oncology Department, Weifang People’s Hospital, Shandong Second Medical University, Weifang, Shandong, China

**Keywords:** tumor-induced osteomalacia, phosphaturic mesenchymal tumor, fibroblast factor-23, soft tissue tumor, hypophosphatemia, popliteal fossa

## Abstract

Tumor-induced osteomalacia (TIO) is a rare paraneoplastic syndrome characterized by hypophosphatemia caused by excessive secretion of fibroblast growth factor-23 (FGF-23) by tumors. This leads to impaired bone mineralization and, ultimately, osteomalacia. The most common underlying cause is a phosphaturic mesenchymal tumor (PMT). Due to its rarity, nonspecific clinical presentation, and limited clinician awareness, TIO is frequently underdiagnosed or misdiagnosed. A 42-year-old man presented with persistent pain in the chest, lower back, knees, and ankles for more than six months, which had worsened in the preceding week. Laboratory tests revealed hypophosphatemia and abnormalities in markers of bone metabolism. Symptomatic treatment provided minimal improvement. The whole-body PET/CT scan subsequently identified a cystic and solid mass in the popliteal fossa of the right knee, with high somatostatin receptor expression. The tumor was surgically removed, and histopathological examination confirmed PMT. The patient’s blood phosphorus concentration returned to normal one week after surgery, and levels of other laboratory indicators gradually returned to normal. Although symptoms persisted during the first postoperative week, significant relief was noted by the second week. This case report highlighted the necessity of improving clinical recognition and management of TIO to ensure timely diagnosis and treatment.

## Introduction

In 1987, Weidner and Santa (1) initially proposed the concepts of phosphaturic mesenchymal tumor (PMT) and phosphaturic mesenchymal tumor mixed connective tissue variant (PMTMCT). Since the partial pathological classification revision by Folpe et al. (2) in 2004, the understanding and diagnosis of tumor-induced osteomalacia (TIO) have significantly advanced. Clinicians have progressively deepened their knowledge of TIO, leading to an increasing number of reported cases. To date, only slightly over a thousand cases of TIO have been documented worldwide (3, 4). Because of its own rarity, non-specific clinical manifestations, reliance on serum phosphate levels from non-routine laboratory tests, and challenges in tumor localization (5), conducting comprehensive and accurate epidemiological studies on tumor-induced osteomalacia remains challenging. Studies demonstrated that the majority of the tumors causing TIO were benign tumors, of which PMT was the main tumor and a few were malignant tumors (6, 7). PMT predominantly occurs in men and is typically small and slow-growing, making its detection challenging (3, 4). It can develop throughout the body, with a predilection for the lower limbs, followed by the head. The prevalence is comparable between soft tissues and bone tissues, although tumors in soft tissues are associated with a higher risk of fractures. TIO is a rare paraneoplastic syndrome primarily caused by tumor overproduction of fibroblast growth factor-23 (FGF-23) (8). Excessive FGF-23 levels can reduce phosphorus reabsorption in the renal tubules and increase phosphorus excretion in the urine, leading to hypophosphatemia. This condition impairs bone mineralization, eventually leading to osteomalacia, characterized by decreased bone density, destruction of bone trabecular structure, and incomplete fractures. Additionally, it results in diminished muscle function (9). This study presented a rare case of PMT-induced TIO located in the popliteal fossa, along with a review of the relevant literature. To date, only three cases of PMT originating from soft tissue in the popliteal fossa have been reported (10, 11).

## Methods

### Case presentation

A 42-year-old man was admitted to our hospital with chest, lower back, knees, and ankles pain for more than half a year, which aggravated for one week. He had been repeatedly diagnosed with osteoporosis and osteoarthritis in other hospitals, and received symptomatic treatments, such as anti-osteoporosis, anti-inflammatory, and analgesic therapies, with unsatisfactory outcomes. The patient had no prior medical history or related conditions. Physical examination indicated remarkable tenderness of the chest ribs, with positive percussion pain in the lower back. The patient experienced pain in the knee and ankle joints during weight-bearing activities. A soft, poorly mobile mass was palpable in the right popliteal fossa, with no significant tenderness or radiating pain to the lower limbs. Muscle strength and tone in the lower limbs were normal.

Laboratory tests indicated decreased levels of serum phosphorus and 25-Hydroxyvitamin D (25(OH)D), while increased levels of alkaline phosphatase (ALP), special sequence of β-collagen, and uric acid. Serum creatinine, thyroid function tests, parathyroid hormone (PTH), and procollagen type I N-propeptide (PINP) were all in normal ranges. Levels of calcium and 24-h urinary phosphorus were normal or slightly decreased (**Table 1**). Tumor marker tests, including TPSA+FPSA, α-fetoprotein (AFP), CEA, CA199, cytokeratin fragment 21-1 (CYF211), carbohydrate antigen 724 (CA724), neuron-specific enolase (NSE), and serum gastrin-releasing peptide precursor, were all normal.

**Table 1:** The outcomes of laboratory tests.

Laboratory Indices Date	3.28	4.4	4.7	4.18	4.28	5.6	5.12	7.6	Normal range
P, mmol/L	0.63	0.42	0.53	0.46	NA	1.04	1.21	1.24	0.8-1.45
ALP, U/L	NA	445	NA	513	409	NA	487	NA	45-125
Ca, mmol/L	2.37	2.11	2.28	2.29	1.97	2.33	2.21	2.32	2.11-2.58
UA, μmo1/L	489	478	506	364	509	NA	457	396	208-428
Cr, μmo1/L	68	73	62	81	63	NA	72	69	51-97
PTH, pmol/L	5.55	NA	NA	NA	NA	NA	NA	NA	1.60-6.90
25(OH)D, nmol/L	61.35	NA	NA	NA	NA	NA	NA	NA	<50 deficient 50-75 insufficient >75 sufficient
β-CTX, ng/mL	1.30	NA	NA	NA	NA	NA	NA	NA	0.1-0.59
PINP, ng/ml	146.3	NA	NA	NA	NA	NA	NA	NA	15.13-58.59
24-h urine Ca, mmo1/24h (24-h urine level, L)	3.86 (1)	4.07 (1.9)	NA	NA	NA	NA	NA	NA	2.5-8
24-h urine P, mmo1/24h (24-h urine level, L)	22.63 (1)	27.93 (1.9)	NA	NA	NA	NA	NA	NA	16-48

“NA” represents that the data does not exist.

**Figure 1:**
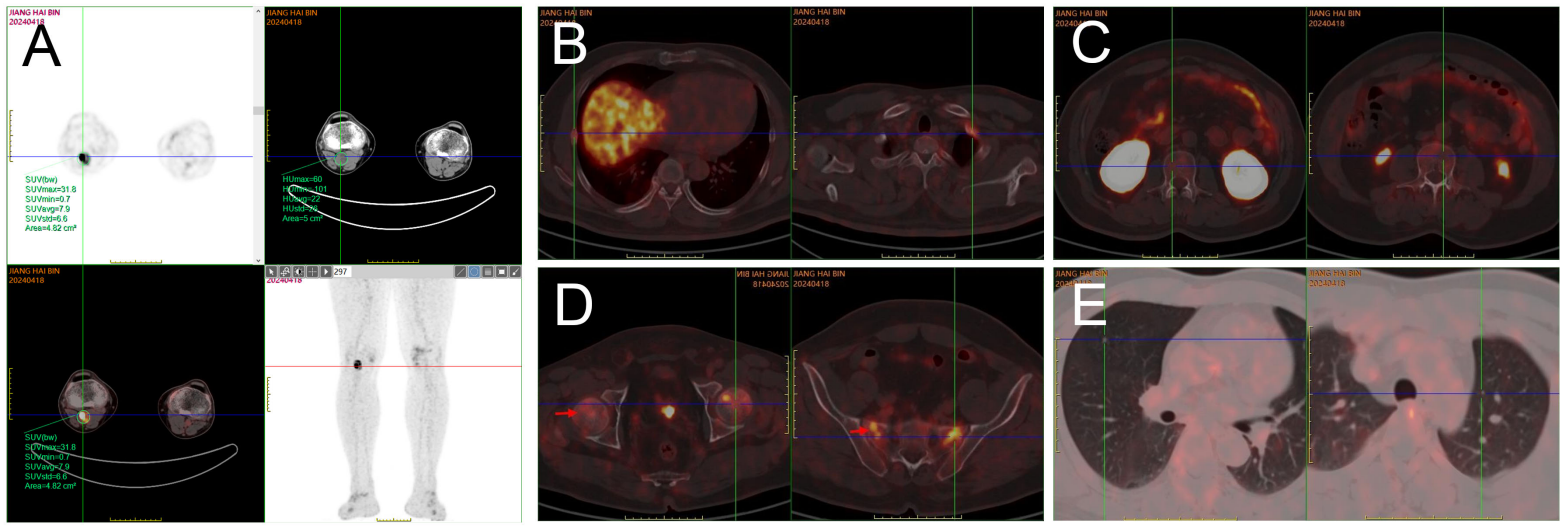
PET/CT whole-body octreotide imaging: After intravenous administration of 18F-octreotide and 70-min resting, whole-body PET/CT tomography was performed. A cystic and solid mass measuring approximately 2.5 cm × 1.8 cm × 3.0 cm with abnormally high radioactivity was detected in the right popliteal fossa, indicating an SUVmax of 31.8 **(A)**. Multiple bilateral ribs exhibited irregular morphology, discontinuous cortical bone, local callus formation, and slightly increased radiotracer uptake, with an SUVmax of 6.0 **(B)**. Flaky areas of increased radiotracer uptake were observed in parts of the thoracolumbar vertebrae, both sides of the sacrum, and bilateral femoral heads, with an SUVmax of 7.2, while no significant bone destruction was evident in the corresponding regions **(C, D)**. Scattered round soft tissue shadows were found in both lungs without significant radiotracer uptake **(E)**.

Imaging examination: Chest computed tomography (CT) showed multiple solid small nodules and nodular-like lesions in both lungs, irregularly shaped multiple bilateral ribs with partial callus formation, and morphological and density changes in multiple thoracic vertebral bodies. Magnetic resonance imaging (MRI) of lumbar vertebra and thoracic vertebra: It revealed mild compression fractures of T3-L5 vertebral bodies, thoracolumbar degeneration, and subcutaneous soft tissue edema in the lumbar region. Whole-body bone imaging: It displayed multiple radioactive foci in bilateral ribs and vertebral bodies, aligning with old fractures based on medical history. A radioactive lesion in the right femoral head suggested the need to exclude aseptic necrosis of the femoral head with further examination. Radioactive concentrations in the bilateral knee joints were primarily considered benign lesions. Positron emission tomography (PET)/CT with whole-body octreotide imaging: 1. It detected a cystic-solid mass in the right popliteal fossa with high somatostatin receptor expression and bilateral rib fractures, consistent with tumor-associated osteomalacia; 2. Slightly increased somatostatin receptor expression was found in some vertebral bodies, the sacrum, and bilateral femoral heads; 3. Multiple small solid lung nodules exhibited no significant somatostatin receptor expression (**Figure 1**). MRI with plain and dynamic contrast enhancement of the right knee joint: It revealed abnormal signal lesion at the medial distal femoral condyle on the right side.

### Surgical treatment

**Figure 2:**
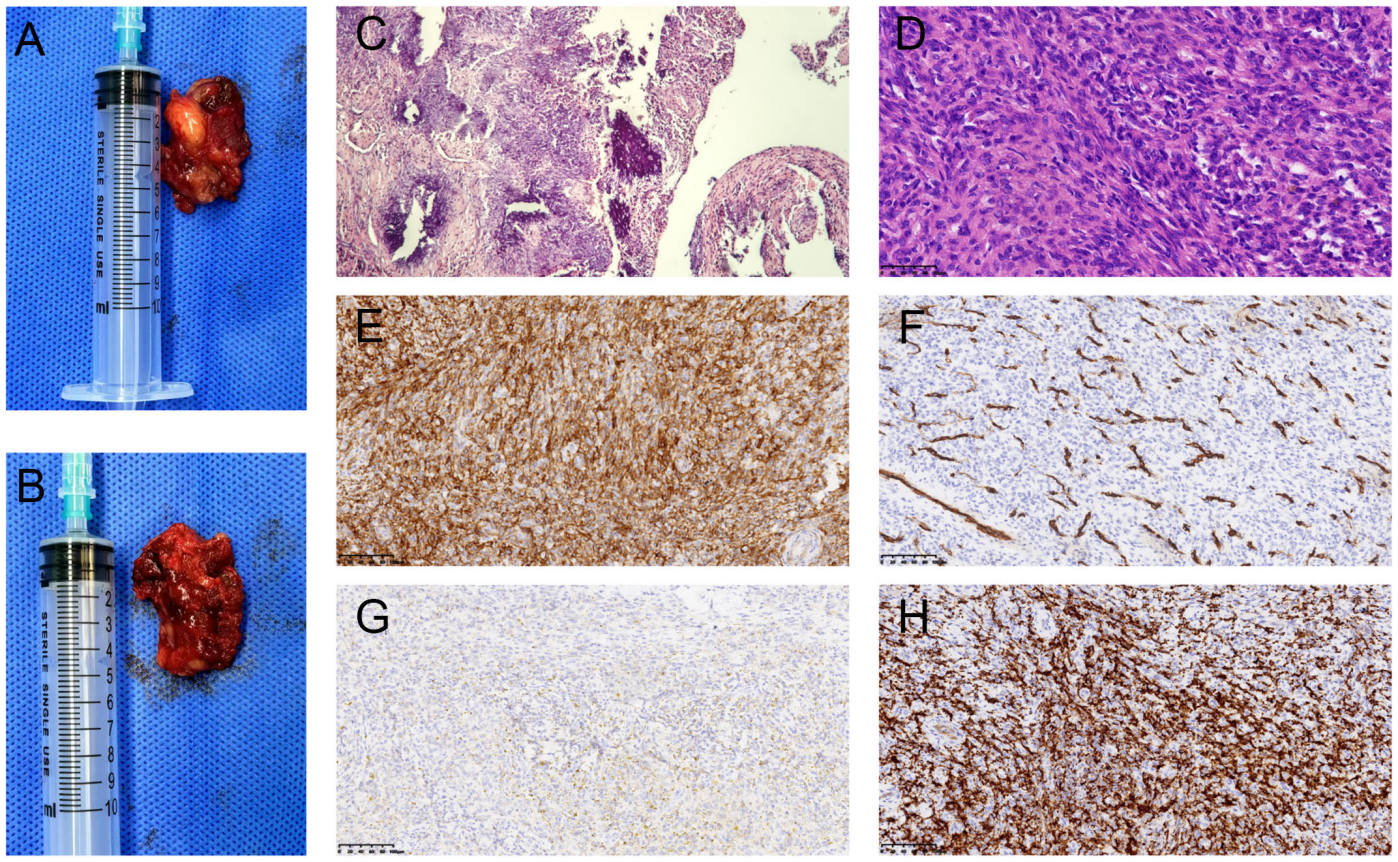
Macroscopic and microscopic views of phosphaturic mesenchymal tumor. Gross examination revealed a grayish-brown tumor with irregular soft tissue and locally cystic on section **(A, B)**. HE staining (×100): The tumor was mainly composed of spindle-shaped cells interspersed with abundant blood vessels of varying thickness, with localized areas showing characteristic flocculent calcification deposition **(C)**. HE staining (×400): Short spindle-shaped tumor cells with no prominent mitotic activity **(D)**. Immunohistochemistry (×200) showed positive staining for SSTR-2 **(E)** and CD34 in blood vessels **(F)**. Macrophage markers CD68 and CD163 also exhibited positive staining **(G, H)**.

The patient was placed in the prone position. After successful general anesthesia, an S-shaped incision was made in the right popliteal fossa, and the skin was cut. The subcutaneous tissues were separated to expose the gastrocnemius muscle. A beige mass was found in the muscular compartment of the gastrocnemius muscle. The mass was soft, well-defined, and surrounded by blood vessels with abundant peripheral blood supply. The blood vessels feeding the mass were ligated, and the mass was bluntly isolated and excised intact (**Figures 2A, B**). The postoperative mass was sent for pathological examination.

## Results

### Pathological findings

The tumor consisted of irregular taupe-colored soft tissue, measuring 3 cm × 2 cm × 1.5 cm. On sectioning, it exhibited local cystic features, with a lumen length of 2.5cm and a wall thickness of 0.1 cm (**Figures 2A, B**). Hematoxylin and eosin (H&E) staining revealed that the tumor tissue was mainly composed of spindle-shaped cells and hyperplastic blood vessels. The spindle-shaped cells were short elongated, with no obvious atypia. The tissue exhibited abundant interstitial blood vessels and focal areas of blue-stained, flocculent calcification (**Figures 2C, D**). Immunohistochemistry revealed the following results: SSTR-2 was positive (**Figure 2E**), blood vessel marker CD34 was positive (**Figure 2F**), and histiocyte markers CD68 and CD163 were positive (**Figures 2G, H**). Some cells exhibited positivity for CD99 and SMA, while scattered cells were positive for S-100. CK and Desmin were negative. The Ki-67 proliferation index was approximately 10%. Pathological diagnosis indicated PMT, located in the right popliteal fossa.

### Follow-up

**Figure 3:**
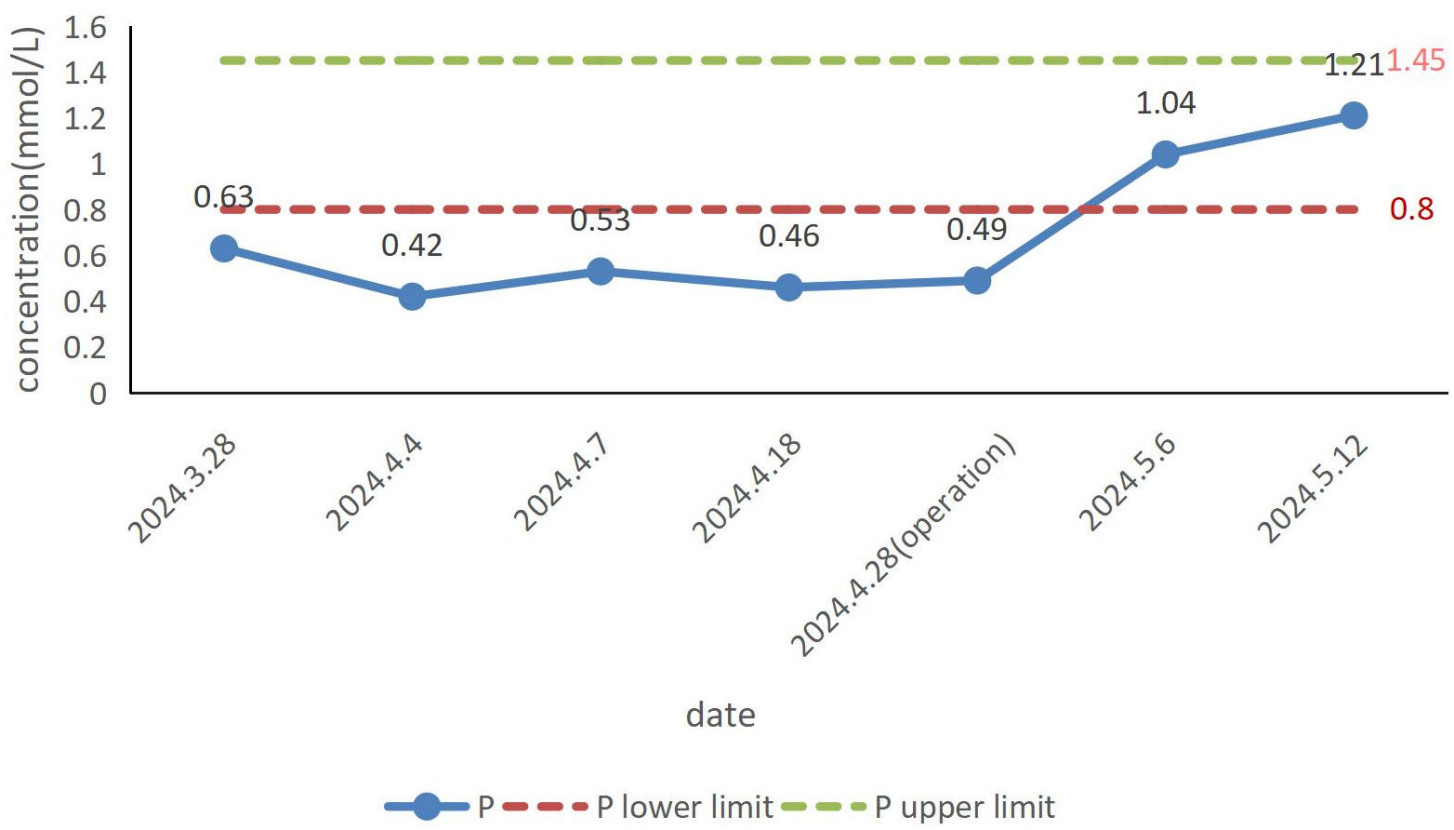
Changes in blood phosphorus concentration in the patient.

The patient’s blood phosphorus concentration had returned to normal one week after surgery (**Figure 3**). Although his symptoms did not fully resolve within the first week after surgery, there was significant improvement by the second week. The tumor was followed up for 3 months, and no recurrence was found.

## Discussion

The clinical manifestation of TIO is primarily related to tumor-induced hypophosphatemia rather than the tumor itself. Because hypophosphatemia can result from various etiologies, the clinical presentation of TIO is non-specific. The most common symptom is bone pain, followed by incomplete fractures and muscle weakness. Over time, the disease may lead to skeletal deformities, such as reduced height, increased thoracic kyphosis, pectus carinatum, and frequent fractures, all of which significantly impact patients’ quality of life (3, 12). Rickets is the main manifestation in children and can be associated with growth retardation. Distal bone pain in the extremities is the most common initial symptom in adults, with ankle and foot being the most commonly affected (12). Muscle weakness is most pronounced in the hip muscles, which is characterized by a faltering gait and marked limitations in activities, such as standing up and climbing stairs. Fractures mainly occur in vertebral bodies, ribs, and femoral neck, resulting in multiple old fractures over time (5, 13). These fractures can lead to reductions in height and increased thoracic kyphosis. Multiple rib fractures may also contribute to the development of pectus carinatum. On physical examination, attention should be given to palpating for a mass, although it is typically not easily detectable due to its small size and slow growth. In the present case, the patient presented with early and significant musculoskeletal pain, bilateral multiple rib insufficiency fractures, multiple mild vertebral compression fractures, and an unremarkable presentation of muscle weakness.

Bone mineralization is a process, in which calcium and phosphorus ions in the body form amorphous calcium phosphate and then deposit into osteoid in the form of hydroxyapatite crystals, leading to normal bone mass. Therefore, when low blood phosphorus is present for a long time, bone matrix mineralization is hindered, resulting in the occurrence of osteomalacia (14, 15). The blood phosphorus level is mainly regulated through kidney reabsorption and intestinal absorption of phosphorus (16). Phosphorus ions are mostly reabsorbed after binding to sodium-phosphorus co-transporters 2a and 2c on the proximal tubules, and FGF-23 inhibits the expression levels of these sodium-phosphorus co-transporters, resulting in the decreased reabsorption of phosphorus by renal tubules, increased excretion, and reduced blood phosphorus (17, 18). Phosphorus ions are absorbed through the intestinal bound phosphate transporter NPT2B, and 1,25-(OH)2D3 can improve its activity. FGF-23 inhibits the synthesis of 1,25-(OH)2D3 by suppressing the activity of kidney 1α-hydroxylase, which in turn reduces the activity of phosphate transporter NPT2B. This ultimately leads to decreased intestinal phosphorus absorption and a reduction in blood phosphorus levels (19). ATP, providing the energy for muscle contraction, is stored in muscle cells as phosphocreatine. Hypophosphatemia reduces the amount of phosphocreatine stored, potentially causing a deficiency in muscle energy (20). In addition, hypophosphatemia results in a decrease in the concentration of 2,3-diphosphoglycerate, which may increase the affinity of hemoglobin for oxygen, thereby reducing oxygen delivery to tissues and leading to tissue hypoxia (21).

Laboratory tests mainly include measurements of serum phosphorus, serum calcium, serum creatinine, ALP, PTH, 25(OH)D, 1, 25(OH)2D, 24-h calcium, urinary phosphorus, tubular maximum resorption of phosphate corrected for glomerular filtration rate (Tmp/GFR), FGF-23, and the percentage of tubular reabsorption of phosphate (%TRP). Blood phosphorus concentration is the most important laboratory parameter. Moderate hypophosphatemia (serum phosphate level, 0.4-0.5 mmol/L) is one of the unique laboratory markers of TIO (22). When the blood phosphorus concentration is within 0.4-0.5 mmol/L, TIO should be suspected. However, since various factors can cause hypophosphatemia, further tests are necessary to determine whether it results from increased renal phosphorus excretion. Tmp/GFR and %TRP are the most accurate parameters for evaluating renal phosphorus consumption and are critical for confirming the diagnosis. In patients with TIO, these values are consistently below the normal reference range (23). FGF-23 concentration is another unique laboratory marker of TIO, with elevated levels found in the majority of patients. However, FGF-23 levels have also been reported to be normal in some TIO cases (23, 24). In addition, laboratory tests in patients with TIO show decreased 1,25(OH)2D levels, typically normal or mildly decreased blood calcium, and normal or elevated PTH. Indicators reflecting osteoclast activity, such as increased ALP, PINP, or specific β-collagen sequences, are often elevated. There may also be increased 24-hour urinary phosphorus and normal or mildly decreased urinary calcium (25). It should be noted that the reduction in levels of 25(OH)D, serum calcium, and urine calcium alone does not confirm TIO. Vitamin D deficiency can also lead to the reduction of 25(OH)D and calcium levels, and its resultant secondary hyperparathyroidism is the most common cause of hypophosphatemia. Therefore, vitamin D levels must be assessed to rule out interference before diagnosing TIO. In this case, most of the patient’s laboratory results were consistent with the typical biochemical markers of TIO, particularly the blood phosphorus levels. These were highly significant, highlighting markedly low blood phosphorus preoperatively and a substantial increase in blood phosphorus postoperatively (**Figure 3**).

There is a systematic method for tumor localization, commencing with a thorough physical examination of the entire body. Although the detection rate through physical examination is low, functional imaging is thereafter used to identify suspicious lesions, followed by anatomical imaging for more precise localization and to clarify the nature of the lesion. To enhance diagnostic accuracy, FGF-23 concentration can be measured using segmental venous blood collection. However, tumors may not always be detected through these methods. According to a retrospective study in Japan (26), the proportion of patients with no tumor found after strict localization method was as high as 37.5%. It should be noted that malignancies causing TIO tumors can metastasize to other parts of the body (6). Weidner et al. (1) identified one TIO patient with a single malignant tumor that originated in the bone, recurred locally, and metastasized to the lungs. In this case, Chest CT revealed multiple small solid nodules and nodular-like lesions in both lungs. After further investigation, these nodules were determined not to be metastases from the tumor.

Functional imaging refers to somatostatin receptor scintigraphy (SRS), a technique that targets somatostatin receptor 2 (SSTR2), which is primarily expressed in tumor cell membranes. This method uses radiolabeled somatostatin analogs to bind to the SSTR2 receptor, allowing for tumor identification. It is typically combined with single-photon emission computed tomography (SPECT) or PET to localize the tumor. Commonly used imaging agents include octreotide, 99mTc-hydrazinonicotinamide (HYNIC)-octreotide, 68Ga-DOTATATE, and fluorine-18 (18F)-AIF-NOTA-octreotide (18F-OC) (27). 18F-OC PET-CT, a novel and highly effective SSR-specific technique, has been exhibited to facilitate accurate localization of the tumor causing the TIO (28), with sensitivity, specificity, and accuracy of 87.5%, 100%, and 88.2%, respectively (29). In this case, 18F-OC was used as an imaging agent to accurately locate the tumor by combining SUVmax values of radioactive foci, CT changes, and the patient’s medical history. Anatomical imaging techniques, particularly contrast-enhanced MRI, were then used to accurately locate the tumor and guide subsequent surgical decisions.

According to the relevant literature, the time delay from the initial onset to tumor-related treatment is 2.5-4 years, with 80% of cases experiencing a delay of over 2 years. In some cases, nearly 100 patients have not had the tumor causing TIO identified despite extensive diagnostic efforts (3, 30, 31). Prior to admitting at our hospital, the patient had undergone treatment at multiple other hospitals, while had not received an accurate diagnosis or appropriate treatment, leading to a delay of over six months from symptom onset to diagnosis.

During the search for the tumor and the wait for surgery following the diagnosis of hypophosphatemia and suspected TIO, the patient received calcium and phosphorus supplementation along with active vitamin D to improve hypophosphatemia and maintain normal calcium and phosphorus metabolism. This treatment also serves as adjuvant therapy after tumor resection to prevent postoperative complications, such as hungry bone syndrome (HBS) (32). FGF-23 levels generally normalize within 24 h following surgery, while blood phosphorus levels return to normal within five days, and clinical symptoms gradually improve (13). Postoperatively, calcium and active vitamin D were routinely supplemented to prevent secondary hyperparathyroidism and HBS, resulting from negative feedback mechanisms (33). For patients with non-localizable, unresectable, persistent, or recurrent tumors, or those who are not candidates for surgery and are opting for conservative treatment, various options are available, including drug therapy, radiotherapy, and guided ablation (9, 23, 34–36). Traditional drug therapy typically includes active vitamin D supplementation and neutral phosphorus solution. However, this approach has drawbacks such as the need for daily monitoring, multiple dosing, and potential side effects, including gastrointestinal intolerance, kidney stones, nephrocalcinosis, impaired renal function, and secondary hyperparathyroidism. Cinacalcet may be considered for patients who are intolerant to phosphate solutions or those with secondary hyperparathyroidism (37). Burosumab is a recombinant fully human IgG1 monoclonal antibody targeting FGF-23 antigen^[44]^, which binds to and inhibits FGF-23 activity, resulting in increased serum phosphorus levels. It has been approved in the European Union, the United States, Japan, and China for the treatment of unresectable/multiple TIOs. In the present case, surgical resection combined with traditional medical treatment resulted in the patient’s blood phosphorus gradually returning to normal, with significant clinical symptom relief.

Further studies are needed to explore the epidemiology and molecular mechanisms of TIO. At present, treatment for TIO primarily targets FGF-23, while additional data are required to support the use of anti-FGF-23 monoclonal antibodies, such as Burosumab or inhibitors of FGF receptor 1-3 (35, 38).

## Conclusions

In conclusion, although TIO is challenging to diagnose and is mainly missed and misdiagnosed, the correct diagnosis can be made successfully if clinicians are familiar with the disease and apply the clinical approach outlined above. Once diagnosed, surgical resection is the preferred treatment.

## Data Availability

The original contributions presented in the study are included in the article/**Supplementary Material**. Further inquiries can be directed to the corresponding author.
