# Supplementary material for: Phosphaturic mesenchymal tumor of the popliteal fossa: a case report and literature review
Source: Front Oncol. 2024 Dec 19;14:1501499. doi: 10.3389/fonc.2024.1501499 (PMC11693668; doi:10.3389/fonc.2024.1501499)
Supplement: Supplementary file 1 [file DataSheet1.docx]

Supplementary Material

# Supplementary Data

Supplementary Material should be uploaded separately on submission. Please include any supplementary data, figures and/or tables.

Supplementary material is not typeset so please ensure that all information is clearly presented, the appropriate caption is included in the file and not in the manuscript, and that the style conforms to the rest of the article.

# Supplementary Figures and Tables

For more information on Supplementary Material and for details on the different file types accepted, please see [here](https://www.frontiersin.org/guidelines/author-guidelines#supplementary-material).

## Supplementary Figures

**Figure 1**


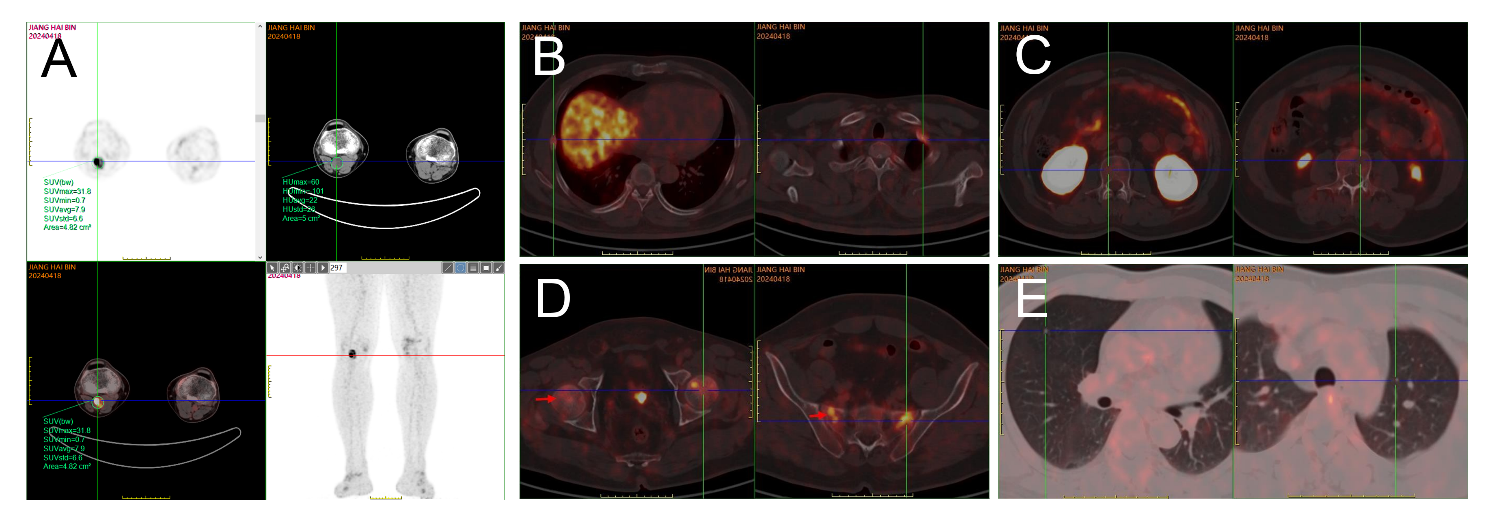
Figure 1. PET/CT whole-body octreotide imaging: After intravenous administration of 18F-octreotide and 70-min resting, whole-body PET/CT tomography was performed. A cystic and solid mass measuring approximately 2.5 cm × 1.8 cm × 3.0 cm with abnormally high radioactivity was detected in the right popliteal fossa, indicating an SUVmax of 31.8 (Figure 1A). Multiple bilateral ribs exhibited irregular morphology, discontinuous cortical bone, local callus formation, and slightly increased radiotracer uptake, with an SUVmax of 6.0 (Figure 1B). Flaky areas of increased radiotracer uptake were observed in parts of the thoracolumbar vertebrae, both sides of the sacrum, and bilateral femoral heads, with an SUVmax of 7.2, while no significant bone destruction was evident in the corresponding regions (Figure 1C and 1D). Scattered round soft tissue shadows were found in both lungs without significant radiotracer uptake (Figure 1E).

**Figure 2**

**
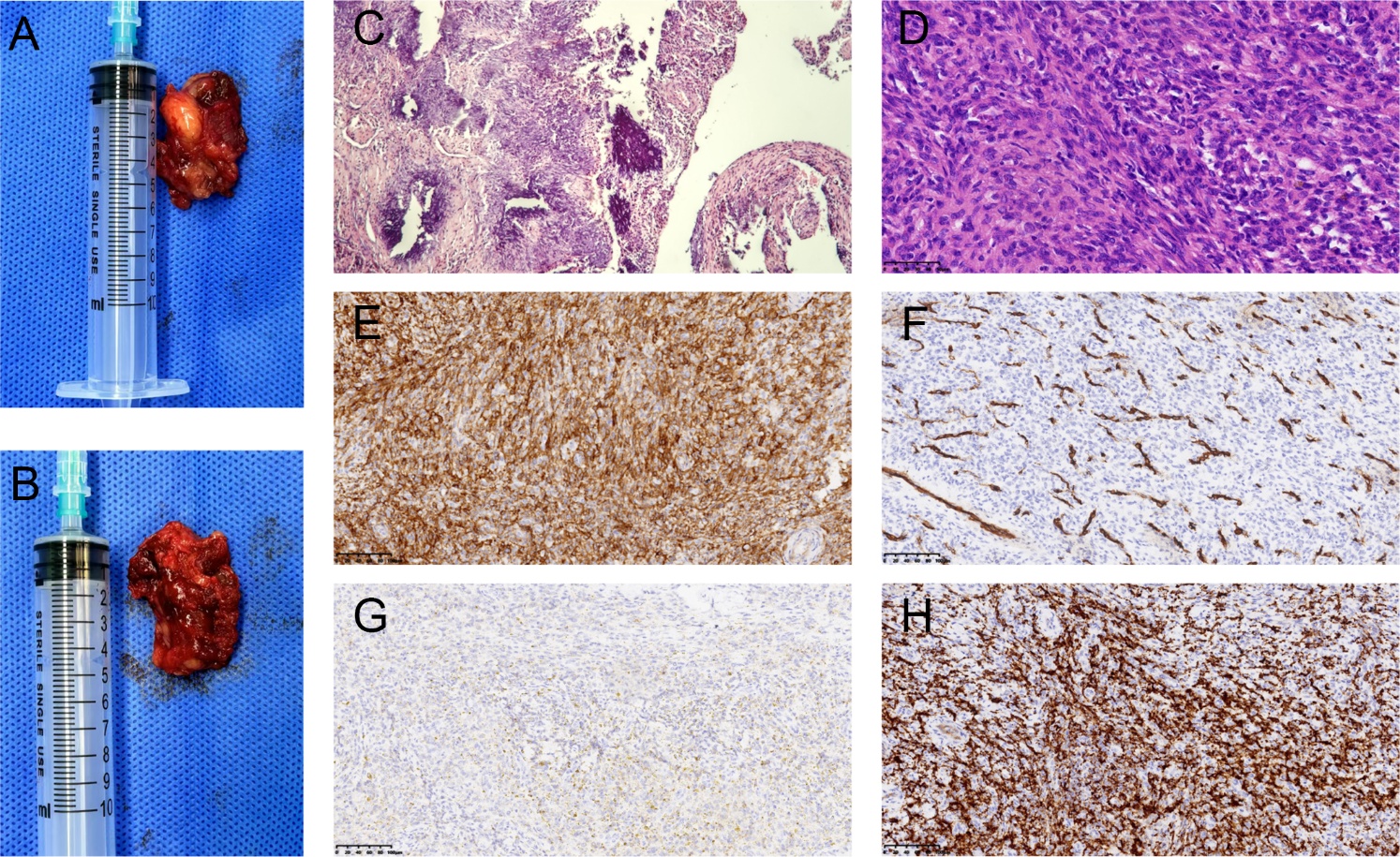
**

Figure 2. Macroscopic and microscopic views of phosphaturic mesenchymal tumor. Gross examination revealed a grayish-brown tumor with irregular soft tissue and locally cystic on section (A and B). HE staining (×100): The tumor was mainly composed of spindle-shaped cells interspersed with abundant blood vessels of varying thickness, with localized areas showing characteristic flocculent calcification deposition (C). HE staining (×400): Short spindle-shaped tumor cells with no prominent mitotic activity (D). Immunohistochemistry (×200) showed positive staining for SSTR-2 (Figure 2E) and CD34 in blood vessels (Figure 2F). Macrophage markers CD68 and CD163 also exhibited positive staining (Figures 2G and 2H).


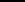


**Figure 3**


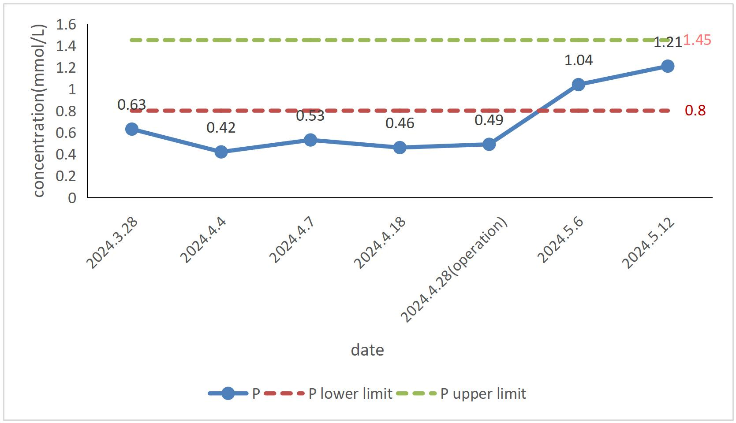


Figure 3. Changes in blood phosphorus concentration in the patient.
